# Ultra-Short Pulses Laser Heating of Dielectrics: A Semi-Classical Analytical Model

**DOI:** 10.3390/ma17215366

**Published:** 2024-11-02

**Authors:** Liviu Badea, Liviu Duta, Cristian N. Mihailescu, Mihai Oane, Alexandra M. I. Trefilov, Andrei Popescu, Claudiu Hapenciuc, Muhammad Arif Mahmood, Dorina Ticos, Natalia Mihailescu, Carmen Ristoscu, Sinziana A. Anghel, Ion N. Mihailescu

**Affiliations:** 1 Faculty of Physics, University of Bucharest, Magurele, 077125 Ilfov, Romaniasanziana.anghel@inflpr.ro (S.A.A.); 2 National R&D Institute for Non-Ferrous and Rare Metals, 077145 Pantelimon, Romania; 3 National Institute for Laser, Plasma and Radiation Physics (INFLPR), Magurele, 077125 Ilfov, Romania; cristi.mihailescu@inflpr.ro (C.N.M.); mihai.oane@inflpr.ro (M.O.); alexandra.trefilov@inflpr.ro (A.M.I.T.); andrei.popescu@inflpr.ro (A.P.); hapenciuc.claudiu@inflpr.ro (C.H.); dorina.toader@inflpr.ro (D.T.); natalia.serban@inflpr.ro (N.M.); carmen.ristoscu@inflpr.ro (C.R.); 4 Intelligent Systems Center, Missouri University of Science and Technology, Rolla, MO 65409, USA; mmahmood@mst.edu

**Keywords:** ultra-short (fs-ps) laser pulses, dielectric targets, analytical modelling, Two Temperatures Model, free electrons density and temperature

## Abstract

Femtosecond laser pulses are currently regarded as an emerging and promising tool for processing wide bandgap dielectric materials across a variety of high-end applications, although the associated physical phenomena are not yet fully understood. To address these challenges, we propose an original, fully analytical model combined with Two Temperatures Model (TTM) formalism. The model is applied to describe the interaction of fs laser pulses with a typical dielectric target (e.g., Sapphire). It describes the heating of dielectrics, such as Sapphire, under irradiation by fs laser pulses in the range of (10^12^–10^14^) W/cm^2^. The proposed formalism was implemented to calculate the free electron density, while numerical simulations of temperature field evolution within the dielectrics were conducted using the TTM. Mathematical models have rarely been used to solve the TTM in the context of laser–dielectric interactions. Unlike the TTM applied to metals, which requires solving two heat equations, for dielectrics the free electron density must first be determined. We propose an analytical model to solve the TTM equations using this parameter. A new simulation model was developed, combining the equations for non-equilibrium electron density determination with the TTM equations. Our analyses revealed the non-linear nature of the physical phenomena involved and the inapplicability of the Beer–Lambert law for fs laser pulse interactions with dielectric targets at incident laser fluences ranging from 6 to 20 J/cm^2^.

## 1. Introduction

Since the advent of lasers in the early 1960s, large efforts have been continuously made for the accurate description of the coherent radiation interaction with solids and liquids [1,2]. The interaction of laser pulses with various materials, such as metals, semiconductors, and dielectrics, has been a critical area of research due to its wide-ranging applications in material processing, microfabrication, and optical engineering. Laser–matter interactions are highly dependent on the material’s optical, thermal, and electronic properties, as well as the laser parameters like wavelength, pulse duration, and energy [3]. For metals, the interaction typically involves linear absorption mechanisms where free electrons in the material absorb the laser energy, leading to thermal excitation and subsequent melting, evaporation, or ablation of the surface. In contrast, semiconductors exhibit a combination of linear and non-linear absorption processes due to their bandgap structure. Laser pulses can excite electrons from the valence to the conduction band, resulting in phenomena like carrier generation, recombination, and melting [4,5]. Dielectric materials, which are transparent to many laser wavelengths under low-intensity conditions, experience a different interaction mechanism. Non-linear absorption processes, such as multiphoton ionization and avalanche ionization, dominate in dielectrics under high-intensity laser irradiation. These materials can undergo rapid electron excitation followed by lattice ionization, leading to material damage or ablation without significant thermal diffusion [6].

Over time, mathematical modelling has diversified and extended from molecular dynamics [7], hydrodynamics [8], Monte Carlo simulations [9], Zhukovsky [10], Finite domain integral transform [11], Fokker-Planck [12] to Finite elements [13] methods. These models consider metals or semiconductors, dielectrics, when subjected to irradiation with shorter and shorter laser pulses. Some of these models are numerical, others are analytical, and many are hybrid. The Two Temperatures Model (TTM), introduced by Anisimov et al. [14], describes the phenomena governing the interaction of short and ultra-short laser pulses with metals, where single-photon absorption and subsequent phonon–electron interactions play a dominant role. In this regard, the study of Guo et al. [15] presents the calculated variations in electron temperature and lattice temperature throughout the process in terms of photon–electron, electron–electron, electron–lattice, and lattice–lattice interactions.

The TTM was extended [16,17] through the appropriate adaptation of target characteristics to account for dielectric properties. The key distinction between metals and dielectrics lies in their bandwidth values, E_g_, which range from 3 to 9 eV for dielectrics, significantly higher than those for metals [18]. As a result, the interaction becomes non-linear, rendering the Beer–Lambert law inapplicable, with multiphoton processes playing the dominant role. A single laser photon, with a wavelength in the visible range (from near IR to near UV), does not provide sufficient energy to excite an electron from the valence band to the conduction band in dielectrics. Therefore, the simultaneous absorption of multiple photons is required to excite a valence-band electron, and the photoionization rate strongly depends on the laser intensity. The required number of photons, *m*, is determined by the relation mħω ≥ E_g_, where ħω represents the photon energy.

Avalanche ionization and the formation of charge traps can also play a significant role, but only at higher laser intensities. A consistent description of laser–dielectric interaction is achievable, and experimental confirmation can be expected if multiphoton absorption, Joule heating, and avalanche ionization are considered [19]. It is also important to note that, unlike in metals, an additional equation must be solved in the case of dielectrics to describe the generation of free electrons. Femtosecond-duration pulses with incident intensities in the range of 10^12^–10^14^ W/cm^2^ were considered in this work, due to the recognized potential for numerous innovative material processing applications. However, the resulting femtosecond laser–material interactions lead to strongly non-equilibrium processes due to the ultra-short irradiation period of a femtosecond laser. As the duration of a femtosecond laser pulse is much shorter than the energy relaxation time of the electron lattice (10^−10^–10^−12^ s), the laser energy absorption is completed before the lattice changes, resulting in a significant non-equilibrium state between electrons and lattices. Heat conduction through the lattice is negligible during the femtosecond pulse duration. As a result, thermal damage (microcracks) and the heat-affected zone are greatly reduced. Due to the non-equilibrium nature of the laser–material interactions, including phase change and material removal, are essentially determined by the laser–electron interactions [20].

It is noted that, under irradiation with pulses of ≤100 fs duration, the generated electrons can induce physical modifications on the target surface. Therefore, ablation should be considered a significant part of the interaction process, occurring in a deterministic manner, unlike the stochastic behavior typical of longer laser pulses [21,22,23,24,25,26,27]. However, the current understanding of fs laser pulse interactions with materials, particularly dielectrics, remains incomplete, despite accounting for absorption, solid excitation, and ablation phenomena. To address this gap, recent theoretical work, based on modifications to the Boltzmann equation [19], has been proposed to offer deeper insights into the non-linear processes governing femtosecond laser pulse interactions with dielectrics [28]. To our knowledge, this paper is the first to develop a simulation model that combines the equations for determining non-equilibrium electron density with the TTM equations. We propose a fully original analytical model considering the solutions of the TTM formalism, and applying the resulting theory to describe the interaction of fs laser pulses with Sapphire. Sapphire was selected for this study due to its unique properties, particularly its exceptional mechanical strength, as well as its heat and electricity conduction characteristics.

## 2. Analytical Model

### 2.1. Solving Free Electron Density

To overcome the inherent challenges in evaluating the temperature field within a dielectric target (such as Sapphire) subjected to laser pulse irradiation (in the fs–ps range), including the continuity of random processes and the validity of mathematical permutations, the proposed approach [16,29] is commonly used to solve the corresponding diffusion equations.

According to the expression derived from the equations in Refs [30,31], and considering multiphoton absorption, avalanche ionization, and the decay of free electrons, the variation law of free electron density in the sample material during heating:

(1) $$\frac{\partial n_{e}}{\partial t}=\alpha_{i}In_{e0}+\delta_{m}I^{m}-\frac{n_{e}}{\tau }$$

The first term on the right side of the Equation (1) is the free electron that is generated by avalanche ionization, the second term represents the free electron generated by multiphoton ionization, and the third term refers to the loss of free electrons caused by inelastic collisions and diffusion from the focal region to the surroundings [17]. We would like to emphasize that the term $n_{e0}$ distinguishes between the free electrons generated by avalanche ionization and those produced by multiphoton ionization. As a result, two major cases have been identified:

(a) $n_{e0}$ = 1 × m^−3^ ≪ $n_{e}$, which is the case reported by Cai et al. [16] and Wang et al. [17], and
(b) $n_{e0}$ = $n_{e}$, which is the case reported by Jiang et al. [30].

The parameters in Equation (1) are collected and defined in Table 1, together with the other parameters used in this work.

In the general form, Equation (1) can be solved with respect to the spatial coordinate *x*, using mathematical methods developed in Ref. [32]. One should note that Equation (2) derives from the assumption of a laser Gaussian beam combined with the Gaussian approximation of the time dependence of the laser intensity in the pulse:

(2) $$I=I_{0}\exp\left[-4ln2{\left(\frac{t}{t_{pf}}\right)}^{2}\right]\exp\left(-\frac{r^{2}}{r_{0}^{2}}\right)$$

Considering the two major cases aforementioned, the final solutions used to describe the free electron-density evolution are written as:

(3) $$n_{e}\left(x,t\right)=\left(\alpha_{i}In_{e0}+\delta_{m}I^{m}\right)\cdot \tau \cdot e^{-\frac{t}{\tau }}$$

for the case $n_{e0}$ = 1 × m^−3^ ≪ $n_{e}$,

(4) $$\mathrm{and}\;n_{e}\left(x,t\right)=e^{\left(\alpha_{i}I-\frac{1}{\tau }\right)t\;}\delta_{m}I^{m}\tau \;$$

for the case $n_{e0}$ = $n_{e}$.

Equations (3) and (4) provide a closed analytical solution [32] that allows for the prediction of free electron-density values in the case of irradiated dielectric target by high intensity, i.e., (10^12^–10^14^) W/cm^2^, fs laser pulses [30]. The typical electron collision time, *t_c_*, is in this case of (1–5) fs. The incident laser intensity in our model is correspondingly given by:

(5) $$I\left(x,z,t\right)=\frac{2F_{f}}{t_{pf}\sqrt{\pi /ln2}}\left(1-R\right)\times \exp\left(-\frac{x^{2}}{r_{0}^{2}}-\left(4ln2\right)\frac{t^{2}}{t_{pf}^{2}}-\int_{0}^{z}\alpha_{abs}dz\right)$$

In Equation (5), the laser absorption coefficient (*α_abs_*) is taken from the reference [17], where the calculation of this coefficient for different values of *z* is presented in detail.

### 2.2. Two Temperatures Model

The TTM is a theoretical framework used to describe laser-heated materials [14]. This model is particularly useful in the study of non-equilibrium phenomena, where the electron and lattice subsystems can be at different temperatures due to various external stimuli, such as laser irradiation or electric fields. The application of TTM is particularly relevant in the study of semiconductors and dielectrics, where rapid heating of electrons can occur without an immediate corresponding increase in lattice temperature [32,33,34,35,36,37]. This separation of temperatures can lead to significant effects on the material’s properties, such as changes in conductivity, optical behavior, and phase transitions.

The TTM formalism was used to describe the interaction between laser radiation and dielectrics. This was achieved by integrating the mathematical techniques from Ref. [38] with the key results from the previous Section. The result is as follows:

(6) $$\left\{\begin{array}{c} C_{e}\frac{\partial T_{e}}{\partial t}=\nabla \left(K_{e}\nabla T_{e}\right)-g\left(T_{e}-T_{p}\right)+Q,\;\\ C_{p}\frac{\partial T_{p}}{\partial t}=g\left(T_{e}-T_{p}\right). \end{array}\right.when\;\left|x\right|\leq r_{0}$$

and,

(7) $$T_{e}\approx T_{p}\approx 0,\;when\;x>r_{0}$$

It follows that:

(8) $$C_{e}\frac{\partial T_{e}}{\partial t}\approx k_{e}\Delta T_{e}-gT_{e}+Q$$

According to Cai et al. [16], the relationship between the two mathematical models is given by the following relation:

(9) $$Q\propto \frac{\alpha_{abs}I}{n_{e}}$$

($\alpha_{abs}$ in the sense reported in Ref. [30]), which, for a given *z*, becomes:

(10) $$Q\left(x,t\right)\propto \frac{F_{f}}{n_{e}\left(x,t\right)}$$

The corresponding Fourier equation is written as:

(11) $$\frac{\partial T_{e}\left(x,t\right)}{\partial t}=\psi \frac{\partial^{2}T_{e}\left(x,t\right)}{\partial x^{2}}+\phi T_{e}\left(x,t\right)\;$$

By application of mathematical techniques inferred in Section 2 with the results in Ref. [38] one obtains:

(12) $$T_{e}\left(x,t\right)=\exp\left(t\phi \right)\frac{C}{2t\sqrt{\pi \psi }}\int_{-\infty }^{\infty }\exp\left(\frac{-{\left(\left.x-\xi \right)\right.}^{2}}{4t\psi }\right)\times \exp\left(-\frac{x^{2}}{r_{0}^{2}}-\left(4ln2\right)\frac{t^{2}}{t_{pf}^{2}}-\int_{0}^{z}\alpha_{abs}dz\right)d\xi$$

with

(13) $$C=\frac{2F_{f}}{n_{e}t_{pf}\sqrt{\pi /ln2}}\left(1-R\right)$$

It should be mentioned that values of Φ and Ψ correlate with Sapphire physical parameters, i.e., −1/τ and thermal diffusivity.

## 3. Model Application and Validity: Sample Cases

We tested the application and validity of the proposed model (for the case $n_{e0}$ = 1 × m^−3^ ≪ $n_{e}$). First, we considered the interaction of an intense laser beam with a Sapphire sample, involving the absorption of six incident photons. The laser beam is assumed to be incident at a point with coordinates x = y = 0, where *z* represents the depth of the Sapphire sample. In the calculations, we used the high-temperature thermal diffusivity value of Sapphire, which is 1 × 10^−6^ m^2^ s^−1^ [39].

Other input data (Tabel 1) were: *λ* = 800 nm, *I* = 4 × 10^13^ W/cm^2^, *σ* = 2.39 × 10^17^ cm^2^, *r_0_* = 4 μm, *R* = 0.95, τ = 150 ps [17], and *E_g_* = 8.8 eV. It should be noted that estimating the absorption coefficient, *α_abs_*, is a major challenge for any model describing Sapphire heating under short-pulse laser irradiation.

According to the literature, there are two main approaches for estimating *α_abs_*: (i) the Keldysh non-perturbative method and (ii) the Wherrett model, which is based on time-dependent perturbation theory. A particular difficulty arises because the *α_abs_* value must be considered zero for the first 0.2 ps of the interaction [39].

Taking these considerations into account, along with the general principles of the semi-classical approach, numerical estimates for *α_abs_* values were introduced as follows:

(14) $$\alpha_{abs}=\left\{\begin{array}{c} 0,\;for\;0<t<0.2\;\mathrm{ps}\\ 1.3\times 10^{6}m^{-1},\;for\;t\geq 0.2\mathrm{ps} \end{array}\right.$$

**Figure 1:**
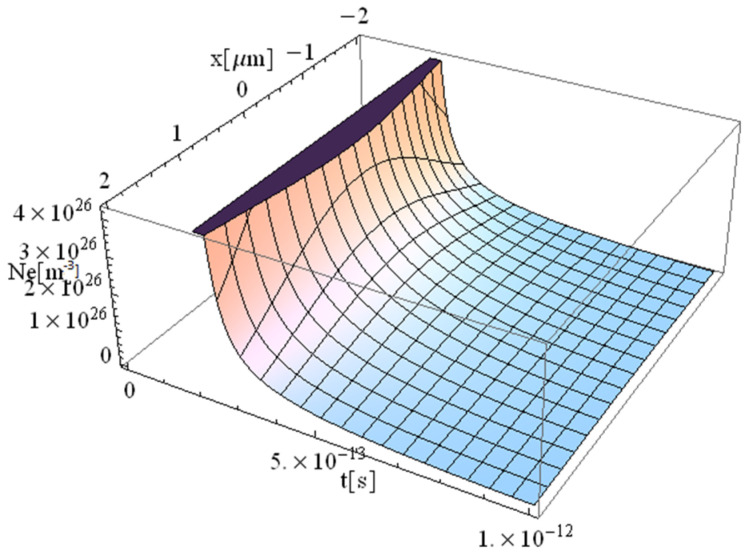
Simulation of a free electron density for a pulse of 225 fs duration incident at the focal spot *y* = *z* = 0.

**Figure 2:**
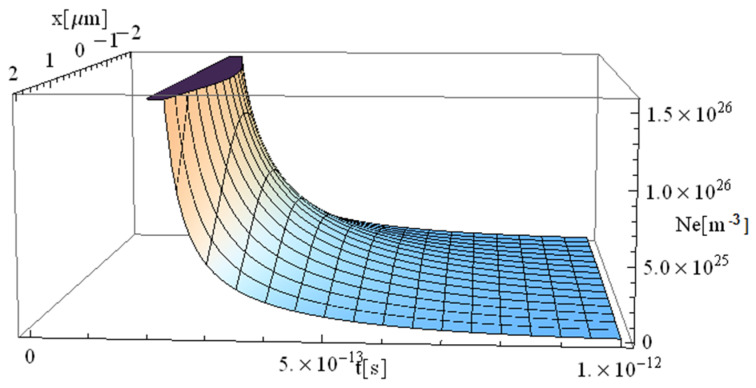
Simulation of a free electron density for a pulse of 225 fs duration at the point of coordinates *y* = 0 and *z* = 1 μm.

**Figure 3:**
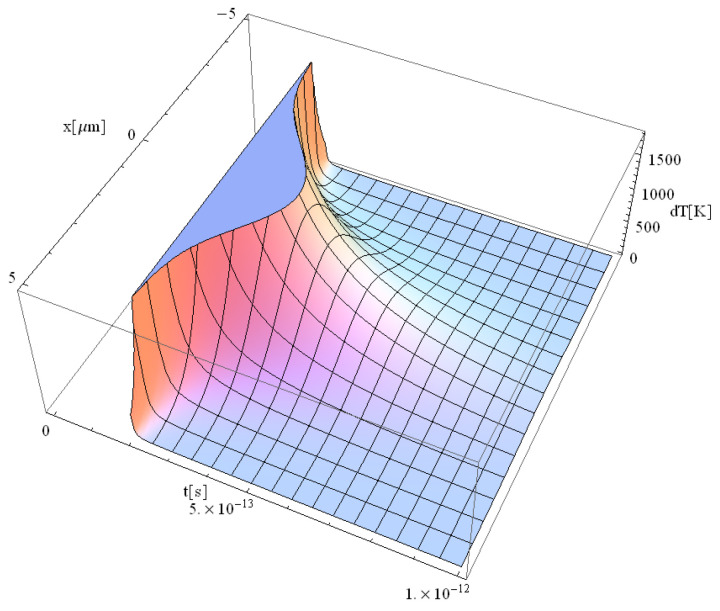
Simulation of a free electron temperature for a pulse of 225 fs duration at the focal spot *y* = *z* = 0.

**Figure 4:**
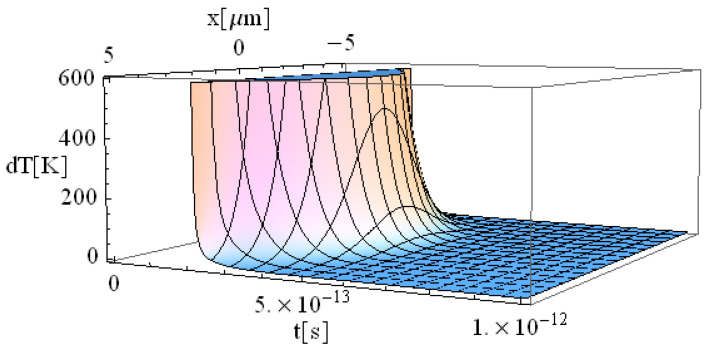
Simulation of a free electron temperature for a pulse of 225 fs duration at the point of coordinates *y* = 0 and *z* = 1 μm.

The obtained results are shown in Figure 1, Figure 2, Figure 3 and Figure 4.

**Figure 5:**
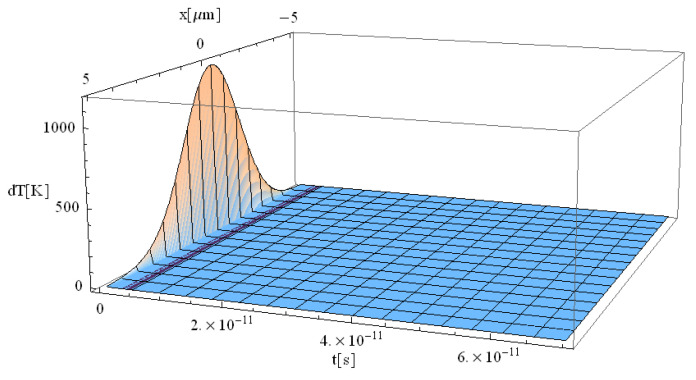
Simulation of a free electron temperature for a laser pulse of 225 fs duration at the point of coordinates *z* = 0.

**Figure 6:**
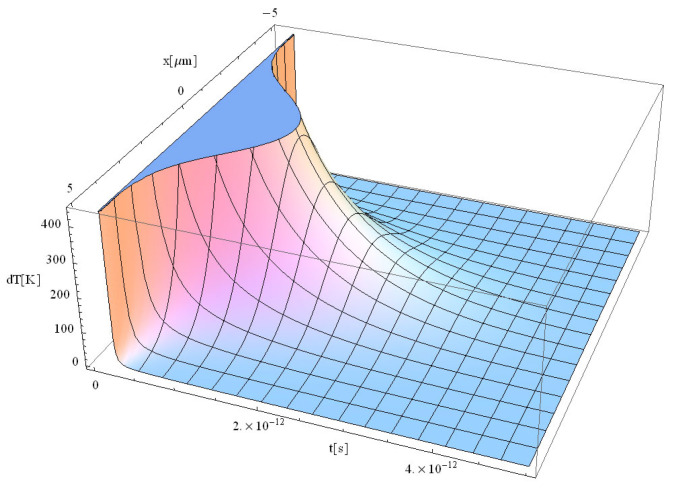
Simulation of a free electron temperature for a laser pulse of 1 ps duration at the point of coordinate *z* = 0.

The significant difference in temperature values between Figure 3 and Figure 4 (for z = 0 vs. z = 1 μm, respectively), can be attributed to the term $\int_{0}^{z}\alpha_{abs}dz$ from Equation (14), which takes on a considerable value, leading to a notable temperature decrease. For comparison, Table 2 presents the values of free electron density (*n_e_*) and temperature (*T_e_*) predicted by our model (M) and those predicted by the model of Cai et al. in Ref. [16]. The data correspond to a 225 fs duration for the proposed model (Figure 1, Figure 2, Figure 3, Figure 4, Figure 5 and Figure 6) and 100 fs for the results in Ref. [16].

There is a good concordance in the free electron-density values, with a variation of (11–25)%, and a smaller deviation in the predicted temperature of (11–16)%. The data indicate that both the electron density and temperature decrease as the penetration depth of the electron beam into the substrate increases. This observation strongly supports the validity of the proposed model, which is significantly faster due to its fully analytical nature. One could draw a parallel with the case of metallic target irradiation using ultra-short lasers by applying the equation in Ref. [40].

The model was subsequently tested regarding the duration of the incident laser pulses. The input data were in this case *λ* = 1030 nm, *α_abs_* = 1.3 × 10^6^ m^−1^, *I* = 6 × 10^13^ W/cm^2^, *σ* = 2.39 × 10^−17^ cm^2^, *E_g_* = 8.8 eV, *r_0_* = 4 μm, and *R* = 0.95. A key distinction is now introduced: the number of absorbed photons involved is eight instead of the previous six. For similar considerations as in the earlier case, we have now used an *α_abs_* value, defined as:

(15) $$\alpha_{abs}=\left\{\begin{array}{c} 0,\;for\;0<t<0.2\;\mathrm{ps}\\ 3\times 10^{7}m^{-1},\;for\;t\geq 0.2\mathrm{ps} \end{array}\right.$$

**Figure 7:**
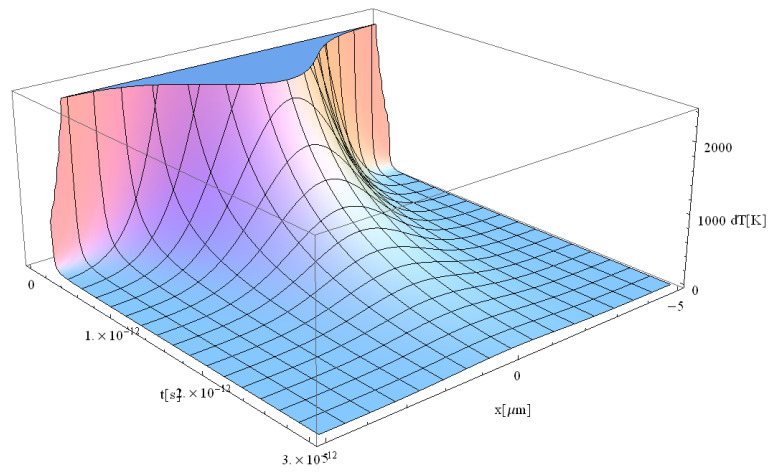
Simulation of a free electron temperature for a laser pulse of 3 ps duration at the point of coordinate *z* = 0.

**Figure 8:**
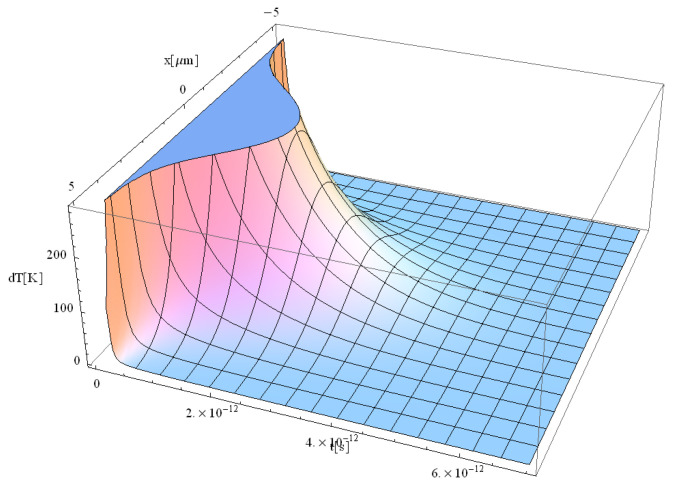
Simulation of a free electron temperature for a laser pulse of 5 ps duration at the point of coordinate *z* = 0.

Typical results of the free electrons temperature are displayed in Figure 5, Figure 6, Figure 7 and Figure 8.

From the study of Figure 5, Figure 6, Figure 7 and Figure 8, the following trends can be observed: (i) the maximum temperature under pulse irradiation increases from the beginning to reach 2000 K for a duration of 3 ps, and (ii) the maximum temperature then decreases for 4 ps to fall to 250 K only for a pulse duration of 5 ps. It should be noted that similar behavior has also been observed in the case of laser irradiation of Si substrates [41].

During the interaction between the laser beam and the dielectrics, in the first approximately 200 fs (Figure 5), the initial free electrons are formed. One should note that no Maxwellian electron gas could be considered, and there exist no defined temperature during the first 200 fs, due to the small number of electrons in non-equilibrium state. After 200 fs (Figure 6), the incident laser interacts with these newly formed free electrons, which allows us to discuss the temperature of the electrons, as demonstrated experimentally by Cai. et al. [16] and Wang et al. [17]. One can therefore speculate that after 200 fs, the electron gas follows a Maxwellian energy distribution function. After approximately 500 fs, the laser begins to interact with the solid (“lattice”) structure of the dielectric [41]. At this point, a new form of temperature, known as lattice temperature, emerges. Additionally, the longer pulse duration in Figure 8 allows more energy to be deposited into the material over time. As a result, the free electron temperature is expected to be higher in Figure 8 compared to Figure 7. This is because the additional 2 ps in Figure 8 give more time for energy absorption, leading to more intense heating. The temperature in ultra-short-pulse laser heating is closely tied to how energy is deposited into the material. A longer pulse duration (as in Figure 8) means that energy is supplied to the electrons for a longer period, increasing their kinetic energy and hence their temperature. One should also note that the energy absorption mechanisms in dielectrics during ultra-short pulses involve non-linear processes such as multiphoton absorption or avalanche ionization. With a longer pulse, these processes have more time to occur, leading to higher lattice temperatures.

The laser width plays a crucial role in determining the spatial electron temperature distribution, and further analysis could provide a more comprehensive understanding of the process.

## 4. Discussion

Recent advancements in the field of ultra-short laser pulses, particularly in the femtosecond (fs) to picosecond (ps) range, have revolutionized the interaction of lasers with dielectric materials. These laser pulses, characterized by extremely short durations and high peak intensities, enable precise control over energy deposition into the material, leading to localized modifications without significant thermal diffusion into surrounding regions [6]. This is crucial in dielectrics like sapphire, a highly transparent and mechanically robust material used extensively in optics, electronics, and industrial applications.

The interaction between fs laser pulses and dielectric targets, including Sapphire, has been previously described by equations that were subsequently solved numerically in the Refs. [42,43]. This demonstrated a good agreement between experimental data and the developed theory of multiphoton processes. The proposed model aims to generate self-consistent analytical solutions via Eq. 11. This direct approach to understanding physical phenomena is made possible because numerical methods (i.e., COMSOL, FEM) [13] can sometimes obscure the underlying physics. The laser–matter interaction, characterized by the photon–electron–phonon energy transfer and free electron evolution, is fundamentally dependent on both the target and the laser beam parameters. In this study, the target is ceramic (i.e., Sapphire), which is typically characterized by its dielectric function, absorption coefficients, and specific heat. Additionally, many experimental investigations have shown efficient epitaxial lateral growth on Sapphire substrates, leading to a reduction in the dislocation density [44].

The parameters characteristic of fs laser pulses, such as wavelength, pulse duration, incident fluence/intensity, and spot waist, govern the interaction with the dielectric and determine the coefficient values for multiphoton absorption, avalanche ionization, and ablation threshold. When the ablation threshold is exceeded, target expulsion is initiated, resulting in plasma generation, a phenomenon that has been extensively studied by N.M. Bulgakova et al. [45].

The decrease in the number of photons absorbed during the multiphoton process is expected to lead to an increase in the depth of penetration into the target material, accompanied by a concomitant decrease in the ablation radius. This evolution aligns with both experimental results and theoretical predictions [16,18], and these features were further confirmed by our simulations. However, it is important to note that in the two experiments analyzed in the previous sections, the laser wavelength was 0.8 μm for six-photon absorption and slightly higher, at 1.03 μm, for eight-photon absorption. This difference contributes to easier and consequently deeper ablation.

This behavior is consistent with our simulations, which predicted a maximum ablation depth of *z* = 0.2 μm for eight-photon irradiations, similar to the data reported in the Ref. [17]. As expected, the maximum penetration/ablation depth increases to 1 μm for the absorption of six photons, which closely aligns with the results presented in the Ref. [16].

It can be assumed that our analytical model is in good agreement with the numerical-analytical models used in the Refs. [16,17], which indicate that an increase in incident intensity leads to non-linear amplification of the laser–target (i.e., Sapphire) interaction. This is evidenced by the emergence and development of multiphoton absorption and avalanche ionization processes. Non-linear phenomena arise for laser incident fluences in the range of (4–6) J/cm^2^, accompanied by significant deviations from the Beer–Lambert law. The ablation depth becomes stationary for fluences above 8 J/cm^2^ [17], which are generally characterized by laser absorption and multiphoton coefficients, reflectivity, and electron-photon coupling factor.

## 5. Conclusions

This work proposes a new analytical model to accurately describe the interaction of femtosecond (fs) laser pulses with dielectrics (i.e., for the specific case of Sapphire), in the range of (10^12^–10^14^) W/cm^2^. For higher intensities, relativistic effects and the possible ”thermal runaway” must be taken into consideration. It should be emphasized that the equations of the proposed model take into consideration the Two Temperatures Method. Thus, the proposed model can be easily extended and adapted for other dielectric materials (which requires the input of specific characteristic parameters/data).

The evaluation of free electron density and the solution of the Two Temperature Models are performed using the differential operator method. The proposed model provides fully analytical solutions, even for ultra-short pulses on the order of fs. This capability enabled us to obtain high-accuracy 3D simulations quickly and easily, which were consistent with predictions from semi-analytical models and confirmed by direct experimental evidence. For instance, using MATHEMATICA software, each simulation required less than one minute on a standard commercial PC with an i7 processor. Overall, simulations with the proposed model demonstrated greater efficiency and significantly faster performance.

The proposed model could be valuable to both experimentalists and theorists, as it is fully analytical and can help describe interference between various non-linear interaction phenomena. This paves the way for innovative processing technologies for traditionally challenging dielectric materials.

## Figures and Tables

**Table 1:** Mathematical symbols used in this work.

Symbol	Name of the Physical Quantity	International Unit of Measurement
*n_e_*	Free electron density	m^−3^
*λ*	Laser wavelength	nm
*τ*	Time constant of electron diffusion	s
*t_c_*	Electrons collision time	fs
*I*	Laser power density	W/m^2^
*α_abs_*	Laser absorption coefficient	m^−1^
*σ*	Avalanche ionization cross-section coefficient	cm^2^
*r*_0_	Laser spot radius	m
*R*	Sapphire surface reflectance	%
*E_g_*	Band gap	eV
*m*	m-multiphoton number	Real number
*α_i_*	Avalanche ionization coefficient	m^2^/J
*δ_m_* (8 photons)	Multiphoton absorption coefficient	m^−3^ s^−1^ (W/m^2^)^−8^
*δ_m_* (6 photons)	Multiphoton absorption coefficient	m^−3^ s^−1^ (W/m^2^)^−6^
*X*	Cartesian Space Coordinate	m
*Y*	Cartesian Space Coordinate	m
*Z*	Cartesian Space Coordinate	m
*T*	Temperature	K
*t*	Time	s
*F_f_*	Laser peak energy density	J/m^2^
*t_pf_*	Femtosecond laser pulse duration	s
*C_e_*	Electron specific heat	J/(K·m^3^)
*K_e_*	Electronic thermal conductivity	W/(m·K)
*Τ_e_*	Electron temperature	K
*T_p_*	Lattice temperature	K
*g*	Electron–lattice coupling coefficient	W/(m^3^·K)
*Q*	Laser thermal fluence	W/m^3^
*Φ*	Fourier coefficient (−1/ τ)	s^−1^
*ψ*	Fourier coefficient (thermal diffusivity)	m^2^/s
*ζ*	Increment real number	Real number

**Table 2:** Correlation between free electron density and temperature values predicted by our model [M] vs. the model reported by Cai et al. [16].

z [μm]	n_e_ [10^26^ m^−3^]	T_e_ [K]	Ref.
0	4	1700	M
1	1.5	600	M
0	4.5	1500	[16]
1	2	500	[16]

## Data Availability

Data included in article/referenced in article.
